# Supplementary material for: Food additive “lauric acid” possess non-toxic profile on biochemical, haematological and histopathological studies in female Sprague Dawley (SD) rats
Source: PeerJ. 2020 Mar 31;8:e8805. doi: 10.7717/peerj.8805 (PMC7120040; doi:10.7717/peerj.8805)
Supplement: Table S1 — Values are expressed as the mean ±standard deviation (n = 6 for each group, female) [file peerj-08-8805-s002.docx]

**Table 1**

The detail classification of the animals

| **Group** | **Treatment** | **Dose** | **Average body weight** | **No. of animals** |
| --- | --- | --- | --- | --- |
| I | Control | ------- | 242.03 ± 4.49 | 6 |
| II | Low dose | 300 mg/kg | 245.51 ± 5.39 | 6 |
| III | High dose | 2000 mg/kg | 243.63 ± 4.72 | 6 |

Values are expressed as the mean ± standard deviation (n=6 for each group, female).
