# Supplementary material for: Food additive “lauric acid” possess non-toxic profile on biochemical, haematological and histopathological studies in female Sprague Dawley (SD) rats
Source: PeerJ. 2020 Mar 31;8:e8805. doi: 10.7717/peerj.8805 (PMC7120040; doi:10.7717/peerj.8805)
Supplement: Table S2 [file peerj-08-8805-s003.docx]

**Table 2**

Dose, route, and frequency of LA in the acute oral toxicity study

| **S.no** | **Agent** | **Diluents** | **Route of administration** | **Frequency of administration** |
| --- | --- | --- | --- | --- |
| 1 | Lauric acid | Tween-20/n/saline | Oral | 300 mg/kg, a single dose |
| 2 | Lauric acid | Tween-20/n/saline | Oral | 2000 mg/kg, a single dose |
